# Switch Function and Pathological Dissociation in Acute Psychiatric Inpatients

**DOI:** 10.1371/journal.pone.0154667

**Published:** 2016-04-28

**Authors:** Chui-De Chiu, Mei-Chih Meg Tseng, Yi-Ling Chien, Shih-Cheng Liao, Chih-Min Liu, Yei-Yu Yeh, Hai-Gwo Hwu

**Affiliations:** 1 Department of Psychology, The Chinese University of Hong Kong, Hong Kong SAR, The People’s Republic of China; 2 Department of Psychology, National Taiwan University, Taipei, Taiwan; 3 Department of Psychiatry, National Taiwan University, Taipei, Taiwan; 4 Department of Psychiatry, Far Eastern Memorial Hospital, New Taipei City, Taiwan; 5 Department of Psychiatry, National Taiwan University Hospital, Taipei, Taiwan; University of New South Wales, AUSTRALIA

## Abstract

Swift switching, along with atypical ability on updating and inhibition, has been found in non-clinical dissociators. However, whether swift switching is a cognitive endophenotype that intertwines with traumatisation and pathological dissociation remains unknown. Unspecified acute psychiatric patients were recruited to verify a hypothesis that pathological dissociation is associated with swift switching and traumatisation may explain this relationship. Behavioural measures of intellectual function and three executive functions including updating, switching and inhibition were administered, together with standardised scales to evaluate pathological dissociation and traumatisation. Our results showed superior control ability on switching and updating in inpatients who displayed more symptoms of pathological dissociation. When all three executive functions were entered as predictors, in addition to intellectual quotient and demographic variables to regress upon pathological dissociation, switching rather than updating remained the significant predictor. Importantly, the relationship between pathological dissociation and switching became non-significant when the effect of childhood trauma were controlled. The results support a trauma-related switching hypothesis which postulates swift switching as a cognitive endophenotype of pathological dissociation; traumatisation in childhood may explain the importance of swift switching.

## Introduction

Dissociation refers to a disruption in the normal integration of consciousness, memory, identity, emotion, perception, body representation, motor control and behaviour (DSM-5, p. 291). Dissociation is the cardinal feature of dissociative disorders and a prevalent comorbidity of several mental disorders including psychotic and mood disorders [1,2]. To investigate the etiology of dissociation, non-clinical and clinical studies have been conducted for two decades. A set of factors linked to dissociation has been identified, despite the ongoing debate on the pathogenesis of dissociation [3–6]. These factors include biological predisposition [7,8], interpersonal adversity [9–11] and neuro-cognitive abnormalities.

Executive control, which is responsible for the manipulation of mental representations and the coordination of mental operations [12], may underlie dissociation [13]. The main effect of dissociation on attention and working memory tasks supports this hypothesis. Studies testing patients with dissociative identity disorder (DID) showed superior disengagement abilities in updating information in working memory and ineffective inhibition about suppressing unwanted mental representations [14–16]. Similar findings were observed in non-clinical dissociative individuals. Non-clinical dissociators showed superior abilities in dividing attention ([17–19]; but also see [20,21] for failures to replicate the finding) and updating information in working memory [22]. Non-clinical dissociators showed ineffective inhibition about suppressing target-related competitors [23,24], task-irrelevant distractors [25] and unwanted mental representations ([26]; but also see [20,21,27–30] for the absence of a dissociation effect on the standard directed forgetting effect and the thought suppression effect). A study using a non-clinical sample also showed that individuals with high dissociation proneness are faster in intra-dimensional switching compared with individuals who display either low or medium dissociation proneness [31].

Empirical studies suggested that executive control is comprised of multiple, separable latent factors [12]. The three core components underlying executive control are the ability to suppress goal-irrelevant distracting representations (inhibition), to discard previous withheld representations (updating) and to shift between competing representations (switching). Whereas studies using non-clinical samples have investigated the link with each of the core executive functions, no study has investigated the relationship between dissociation and switching in a clinical sample. We postulate that swift switching among mental representations leads to disintegrated representations and, hence, plays a vital role in the neuro-cognitive etiology of pathological dissociation. As pathological dissociation is linked to traumatisation [11], we posit a *trauma-related switching* hypothesis. We expect that patients displaying more symptoms of pathological dissociation will be better at switching between different aspects of mental representations and traumatisation can explain this relationship. To our knowledge, no prior study has investigated the intertwined relationship linking traumatisation, switching and pathological dissociation.

The aim of this study is to verify the hypothesis in psychiatric inpatients. Rather than focusing on a specific disorder, a clinical sample of unspecified psychiatric inpatients was recruited for important reasons. Firstly, pathological dissociation appeared to be common, co-morbid psychopathology which is under-reported in clinical practice. Eighty-three percent of dissociative pathology was not accurately recognised in clinical practice [32–37]. Secondly, measuring dissociation across various mental disorders can reduce range restriction so that the conclusion is not limited to a specific pathology.

Cognitive tasks measuring inhibition, updating, switching and intellectual function were included in the assessment. We examined whether swift switching was fundamental to relate pathological dissociation to executive control and whether this link was independent of intellectual function. Furthermore, we verified whether traumatisation played a critical role in relating pathological dissociation to swift switching, by measuring childhood and adulthood trauma. General psychopathology including anxiety-depression, paranoia-psychosis and panic-phobia was also measured as dissociative pathology is often embedded in poly-symptomatic complaints [38,39].

## Methods

### Participants

This study is part of a clinical project on dissociation in acute psychiatric inpatients; the details about the source of participant recruitment can be found elsewhere [9]. The investigation was carried out in accordance with the latest version of the Declaration of Helsinki. The project was approved by the Institutional Review Board at the National Taiwan University Hospital. Participants were recruited from two acute wards. All participants, upon their agreement, were referred to the principal researcher. Eighty-nine participants were approached. Of all participants, 73% percent were female and the averages of age and education were 36±12 years old and 12.8±2.7 years. Primary clinical diagnoses of these participants included psychotic disorders (45%), bipolar affective disorders (26%), depressive disorders (17%), eating disorders (9%) and others (3%). All of the primary clinical diagnoses were made by the caring medical teams according to the DSM-IV-TR. Nine participants did not complete the assessment because of their unstable mental status. Data from 80 participants were used in the analysis.

### Instruments

Three self-report scales were used to evaluate dissociative symptoms, general psychopathology and potential traumatising events. Dissociation was assessed by a validated Mandarin version of the Traumatic Dissociation Scale (TDS; Carlson and Waelde, unpublished results; Cronbach’s alpha = .93). The TDS consists of 24 items on five dimensions of pathological dissociation, including gaps in awareness, gaps in awareness and re-experiencing, depersonalisation, derealisation and amnesia. The frequency of each item within the past week is rated on a 5-point Likert’s scale from 1 (never) to 5 (more than ten times). A total score is used as an indicator of the degree of pathological dissociation. General psychopathology was assessed by a validated Mandarin version of the Symptom Check List-90-Revised (SCL-90-R; Cronbach’s alpha = .98) [40]. The SCL-90-R contains 90 items, with nine subscales including somatization, obsession-compulsion, interpersonal sensitivity, depression, anxiety, hostility, phobia-anxiety, paranoid ideation and psychoticism. An explorative factor analysis in psychiatric patients reported a three-factor solution including anxiety-depression, paranoia-psychosis and panic-phobia [39]. Each item is rated on a 5-point scale from 0 (not at all) to 4 (extremely). A total score and three subscale scores were used in the analysis. Potentially traumatising events were evaluated by a validated Mandarin version of the Brief Betrayal Trauma Survey (BBTS; Cronbach’s alphas = .76 and .79 for the childhood and adulthood subscales, respectively) [23,41]. The BBTS consists of twelve potentially traumatising events (e.g., sexual, physical or emotional abuse) inflicted by close and non-close others before 18 years of age (childhood trauma) and after (adulthood trauma). Each experience is rated on a 3-point scale (never, once to twice and more than twice) and converted into a dichotomous variable with a value of 0 (no) and 1 (yes). Two subscale scores (childhood and adulthood trauma) are obtained.

### Cognitive Tests

*Wechsler Adult Intelligence Scale*, *the third version (WAIS-III*)[42]. The original WAIS-III comprises of 13 subtests; the full test requires 90 minutes to complete. In order to save time and obtain a gross estimate of intellectual function, a short form consisting of information, block design, arithmetic calculation and digit symbol substitution was used [43,44]. These four subtests assessed the functions of the four dimensions underlying the original 13 subtests.

*Switching*. A typical set-switching task requires participants to perform two distinct and competing tasks on the same stimulus set (e.g., A and B tasks) [45]. In the baseline condition, the two tasks are separate. Participants perform one task first and then the other task. In the switch condition, the two tasks are mixed trial by trial. Participants engage in the two tasks successively, doing the A task first and then the B task and shifting back to the A task (e.g., ABAB). Alternation in the two task sets requires terminating the operation mode of the previous task and reconfiguring the competing mode for the upcoming task [46,47]. The increase in the reaction time for switching (switch cost: the reaction time of the switch condition–that of the baseline condition) serves as a switch index. Smaller switch costs indicate higher efficiency in switching. Individuals with smaller switch costs have a better ability to switch between the mental operations.

Two classification tasks that require participants to process different aspects of mental representations were used in this study [48]. The first task was to judge whether each object was living or non-living. The second task was to compare whether each object was bigger or smaller than a football. Twenty items which can be classified on the basis of both rules were selected. Half of the items were living, and half of them were bigger than a football. In the baseline condition, participants focused on the animacy judgment in the first block, and performed the size judgment in the second block. In the switch condition, participants continuously alternated between the two tasks in two consecutive blocks. In the first block, ten items were used for animacy judgment and ten items were used for size judgment. In the second block, the items used for animacy judgment in the first block were used for size judgment, and vice versa.

*Random Number Generation (RNG)*. Participants were instructed to randomly generate an Arabic number, any integer from 1 to 10, every 1.5 second. A random sequence would not contain adjacent number values (e.g., 78, 54), a preponderance of repetitions (e.g., 22, 919) and a stereotyped sequence (e.g., 135, 246, 963). The instruction adopted for children was used for easy comprehension [49]. The generation of random responses requires successively monitoring current outputs, suppressing stereotyped sequences, and concurrently conducting some addition/deletion which requires executive control [50]. Ten trials were practised before the task began. A total of 110 responses were collected. The responses were analysed with a computerised program and 16 behavioural indicators were derived [51].

The factorial structure of these behavioural indicators has been investigated. Similar three-factor solutions with prepotent associates, equality of response usage and repetition avoidance were reported [48,49,51,52], although the indicators of each factor were slightly different. Using structured equation models, the relationship between the three factors and other executive control tasks was examined [48]. The results showed that, except for repetition avoidance, the other two factors were associated with executive control. Prepotent associates were related to inhibition and equality of response usage was related to updating. Repetition avoidance may be pertinent to automatic processes.

To validate the generalizability of the three-factor solution in a clinical sample, exploratory factor analysis was conducted. The principal factor method was used for factor extraction, the parallel analysis for the determination of the number of factors, and the promax method for rotation. A three-factor solution similar to those reported from the non-clinical samples was obtained (See S1 Table for the factor loadings). The three most reliable indicators across the current and past studies of prepotent associates and equality of response usage were selected. These behavioural indicators were standardised and summed, respectively, to form an inhibition index (RNG, TPI, Adjacency and Runs) and an updating index (R, Repetition Gap Mean and Repetition Gap Median).

### Procedure

Written informed consent was obtained after the procedure was fully explained. The assessment was conducted individually in a quiet testing room in each ward. The assessment started with the short version of the WAIS-III, followed by the random number generation task and then the switching task. The self-report measures were then administered.

## Results

We examined whether our measures replicate previous findings that showed interrelated and separable executive functions using correlation analysis with Pearson’s product-moment coefficients. A significant correlation with a medium effect size was observed between the switching and inhibition indices and non-significant correlations were observed between these two indices and the updating index [r_Inhibiton&Switching_(80), r_Inhibition&Updating_(80) and r_Switching&Updating_(80) = 0.29, -0.17, and -0.08; Ps = 0.01, 0.15 and 0.49]. The lack of high correlations among the three indices supports the notion that the three components of executive control are separable [12]. To investigate whether intellectual function can be confounding for relating executive control and dissociation, we examined the correlation between each executive function and intellectual function. The switching and inhibition indices negatively correlated with the IQ score, indicating that higher intellectual function was linked to better switching (less switch cost) and inhibition (fewer prepotent associates) performance; non-significant correlation was found between the updating index and the IQ score [r_IQ&Switching_(80), r_IQ&Inhibition_(80) and r_IQ&Updating_(80) = -0.38, -0.37 and 0.15; Ps = 0.0007, 0.001 and 0.21]. Given the significant correlations, intellectual function was controlled in latter regression analysis.

We then examined whether the cognitive measures can discriminate between patients with and without pathological dissociation in this clinical sample. A thorough analysis targeting the intertwined relationship between dissociative symptoms, general psychopathology and trauma history can be found elsewhere [11]. Table 1 shows the descriptive statistics of the self-report measures and cognitive performance in patients with high or low (median-split) TDS scores. Pearson’s product-moment correlation coefficient was then applied to delineate the association between pathological dissociation and cognitive performance. Using correlation analysis can better preserve information that may be lost in the group comparison [53]. A distinct profile on executive control was observed, depending on the TDS scores. The TDS total score correlated significantly with the switching and updating indices [r_TDS&Switching_(80) and r_TDS&Updating_(80) = -0.27 and 0.23; Ps = 0.02 and < .05]. This finding indicates that a higher score of dissociation was linked to better performance on switching (less switch cost) and updating (larger gaps between repeated responses). No significant correlation was found between dissociation and the inhibition index [r_TDS&Inhibition_(80) = 0.01, P = 0.91]. More importantly, the TDS score did not correlate significantly with the measures of intellectual function [r_TDS&IQ_(80) = 0.02, P = 0.86] and performance measure in the baseline condition of the switching task [r_TDS&Baseline_(80) = 0.02, P = 0.86]. That is, patients with pathological dissociation showed comparable performance in the tasks that did not target executive control.

**Table 1:** Descriptive statistics of the behavioural and self-report measures between inpatients with high and low pathological dissociation.

		High TDS		Low TDS	
		M	SD	M	SD
Traumatic Dissociation Scale					
	Total score	55.5	13.94	26.01	1.94
	Depersonalisation	9.5	3.53	5.14	0.36
	Derealisation	10.9	4.33	5.08	0.3
	Gap in awareness (GA)	13.7	3.74	5.75	1.11
	Amnesia	8.33	3.13	3.36	0.83
	GA and re-experiencing	13	3.72	6.68	1.22
Symptom Check List-90-revised					
	Total score	1.83	0.8	0.66	0.67
Brief Betrayal Trauma Scale					
	Childhood trauma	3.31	2.95	0.94	1.47
	Adulthood trauma	3.17	2.83	0.86	1.27
WAIS-III (a short form)					
	Intellectual quotient	95.3	14.41	93.42	15.53
Set switching task					
	Baseline condition	67.4	24.31	85.91	51.99
	Switch condition	137	55.72	183.4	122.21
	The switch index	69.6	47.34	97.49	80.44
Random Number Generation task					
	The updating index	3.01	0.62	2.6	0.69
	The inhibition index	-0.2	0.51	-0.04	0.69
	Repetition avoidance	0.04	0.69	0.13	0.91

Hierarchical regression analysis was carried out to verify 1) whether the correlations with executive control remained significant when the effects of demographic variables and intellectual function were considered, and 2) whether switching was the vital function relating to pathological dissociation. There were two steps in the analysis. First, a baseline model was constructed with the TDS total score as a dependent variable and the IQ score and the demographic variables including gender, age and years of education as the predictors. Furthermore, a second model was constructed by adding the three indices of executive control as predictors. The incremental coefficient of determination (△R^2^) between the baseline model and the Step 2 model is important. Table 2 shows the results. The three measures independently explained 11% of the variance of the TDS total score [partial F test, F*(M_demographics&IQ_|M_demographics&IQ&executive control_) = 4.48, d.f. = 3, P < 0.05]. More importantly, switching rather than updating was the function that showed a significant relationship with pathological dissociation.

**Table 2:** Regressing pathological dissociation with demographical variables, intellectual quotient, executive control and traumatic experience in hierarchical regression analysis.

Model	Predictors	Beta	T	P
Baseline				
	Gender	0.25	2.18	0.03
	Age	-0.02	-0.20	0.84
	Education	-0.20	-1.41	0.16
	Intelligence	-0.09	-0.14	0.52
	[Model testing: F = 1.66, P < 0.17, R^2^ = 0.09]			
Step 1				
	Switch index	-0.37	-2.11	0.04
	Updating index	0.22	1.56	0.13
	Inhibition index	0.02	0.18	0.86
	[Model testing: F = 2.19, P = 0.048, R^2^ = 0.20]			

The roles of trauma history and general psychopathology were explored. Partial correlations were used to examine the correlations with the switching index after removing the effects of potentially traumatising events and comorbid psychopathology. The correlation became non-significant after partialling out the effect of childhood trauma rather than adulthood trauma [for childhood and adulthood trauma, rs = -0.19 and -0.28, Ps = 0.12 and 0.02, respectively]. Regarding the covariate effect of comorbid pathology, the correlation also became non-significant after partialling out the effect of general psychopathology [r = -0.03, P = 0.82]. Similar results were observed when using the three subscale scores in the analysis [for anxiety-depression, paranoia-psychosis and panic-phobia, rs = -0.01, -0.12 and -0.12, Ps = 0.92, 0.34 and 0.32, respectively].

## Discussion

The aim of this study is to verify a trauma-related switching hypothesis of pathological dissociation. We expect that switch function is associated with pathological dissociation across various mental disorders and the link would be associated with traumatisation. Consistent with our prediction, the results from a regression analysis showed that switch cost significantly predicted pathological dissociation beyond demographic variables and intellectual function whereas updating and inhibition abilities did not. More importantly, this relationship became non-significant when the effect of childhood trauma was controlled.

Our results showed that acute psychiatric inpatients who displayed more dissociative symptoms performed better than those with less dissociative symptoms at updating and switching. The enhanced capability of these two executive functions may appear counter-intuitive as patients with high dissociative symptoms also displayed complex psychopathology [38,39]. Yet, the results are consistent with the findings that dissociators are better at switching attention between perceptual attributes and updating information in working memory in non-clinical samples and clinical patients with DID [16,22,31]. The findings extended the previous findings from DID patients to patients diagnosed with other psychiatric disorders. Superior cognitive control may underlie the characteristic over-modulation of emotional representations under catastrophic stress in traumatised patients comorbid with dissociative pathology [54,55].

The non-significant correlation between pathological dissociation and inhibition seems inconsistent with the finding of weakened inhibition in non-clinical and clinical dissociators [14,15,22–26]. This inconsistency may have arisen because inhibition involves multiple mechanisms at different levels of cognitive processes [52,56–58]. In fact, non-significant results were also reported from studies using other paradigms such as thought suppression [29,30]. It is possible that the inhibitory mechanism involved in the random number generation task is different from the inhibitory mechanisms engaged in the tasks used in prior studies. Systematic research is required to investigate the relationship between pathological dissociation and various inhibitory mechanisms.

The critical finding is the result of the regression analysis. Switching remained significant when all the three components of executive control were considered. Although the correlation between pathological dissociation and updating might have become significant if the sample size had been larger, the non-significant result implies that the superior ability to update mental representation may not be the most critical component of executive control underlying dissociation. Swift switching, which may lead to disintegration of mental representations, plays a vital role in the etiology of pathological dissociation. Whether swift switching leads to the compartmentalisation of self-related representations, such as autobiographic memory in clinical dissociators, remains for future research to investigate.

Consistent with our hypothesis, trauma plays an important role in linking pathological dissociation and swift switching. When the effect of childhood traumatisation was controlled statistically, the correlation between dissociation and switching became non-significant. We cannot, however, conclude whether swift switching results from traumatisation, because of the retrospective nature of the study. Future research should clarify the intertwined relationship between dissociation, switching and traumatisation. It is noteworthy that the result is in contrast to the findings from several non-clinical studies which showed that potentially traumatising events in early developmental stages could not explain the atypical executive control in non-clinical children and adults with dissociation proneness [18,23,24]. There may exist distinct patho-genetic trajectories of pathological and non-pathological dissociation. Yet, superior cognitive disengagement appears to be an endo-phenotype of dissociation in both non-clinical and clinical dissociators.

It was noted that the association between pathological dissociation and swift switching also became non-significant when the effect of general psychopathology was removed. The overlap among superior disengagement abilities, pathological dissociation and other psychopathology was consistent with the study of patients with dissociative identity disorder [16]. Interpretation of the overlap between dissociation and general psychopathology is difficult as patients with pathological dissociation usually present with a complex profile of comorbid psychopathology including anxiety, mood symptoms, somatization, and even psychotic-like experience [38,39]. General psychopathology may be considered as comorbidities of dissociation with common biological or environmental risk factors, as an associated feature of dissociation, or as a secondary reaction to dissociation [4]. More studies are required to clarify this puzzling finding.

For clinical implications, swift switching may enable patients with pathological dissociation to avoid intrapsychic conflicts and to reduce the accessibility of unwanted memories. This cognitive avoidance may lead to short-term relief from distress by diverting attention from unwanted stressful materials to less threatening stimuli. However, swift switching may disrupt the formation of inter-relations among representations, contributing to the inefficient inhibitory control over competing representations [23]. Swift switching to a new mental set may also impose an interference effect on the activation of target representations [24,59] and even the associations between target representations and contextual details [60], leading to unusual forgetfulness of autobiographical experience. Autobiographical memory thus may be poorly regulated, preventing the formation of coherent and stable self-knowledge [61]. Detecting switching behavior within a clinical session may be crucial to understand a client’s internal conflicts related to the change and beneficial to treatment [62,63].

### Limitations

In addition to the inability to show the trajectory that links trauma and swift switching with pathological dissociation and psychopathology, the impact of medication on superior switching remains unclear. Given the poly-symptomatic nature of pathological dissociation, different medications may be prescribed for dissociative and non-dissociative patients [38,39]. Nevertheless, we consider that medication plays a minimal role in linking pathological dissociation and switching because non-clinical individuals with dissociation proneness also show swift switching.

## Supporting Information

S1 Table Factor loadings of the behavioural indicators of the random number generation task. (DOC)
