# Supplementary material for: Switch Function and Pathological Dissociation in Acute Psychiatric Inpatients
Source: PLoS One. 2016 Apr 28;11(4):e0154667. doi: 10.1371/journal.pone.0154667 (PMC4849636; doi:10.1371/journal.pone.0154667)
Supplement: S1 Table — (DOC) [file pone.0154667.s001.doc]

S1 Table

Factor loadings of the behavioural indicators of the random number generation task.

| Measures |  | Factor1 |  | Factor2 |  | Factor3 |
| --- | --- | --- | --- | --- | --- | --- |
| Random Number Generation |  | 0.67 |  | -0.31 |  | -0.22 |
| Turning Point Index |  | -0.84 |  | -0.12 |  | 0.09 |
| Adjacency |  | 0.81 |  | 0.03 |  | 0.14 |
| Runs |  | 0.85 |  | 0.19 |  | 0.00 |
| Redundancy |  | 0.02 |  | -0.90 |  | -0.06 |
| Mean Repetition Gap |  | -0.05 |  | 0.96 |  | -0.20 |
| Median Repetition Gap |  | 0.18 |  | 0.53 |  | -0.18 |
| Phi 3 |  | 0.16 |  | -0.07 |  | 0.89 |
| Phi 4 |  | -0.02 |  | 0.07 |  | 0.71 |
| Phi 5 |  | -0.10 |  | -0.22 |  | 0.51 |
| Phi 6 |  | -0.17 |  | -0.16 |  | 0.48 |
| Random Number Generation 2 |  | 0.42 |  | -0.47 |  | -0.31 |
| Coupon |  | -0.01 |  | -0.03 |  | 0.01 |
| Mode Repetition Gap |  | 0.10 |  | 0.14 |  | -0.29 |
| Phi 2 |  | 0.35 |  | 0.30 |  | 0.45 |
| Phi 7 |  | -0.11 |  | -0.35 |  | 0.35 |
